# A Novel Gesture-Based Control System for Fluorescence Volumetric Data in Virtual Reality

**DOI:** 10.3390/s21248329

**Published:** 2021-12-13

**Authors:** Vratislav Cmiel, Larisa Chmelikova, Inna Zumberg, Martin Kralik

**Affiliations:** Department of Biomedical Engineering, Brno University of Technology, Technicka 12, 616 00 Brno, Czech Republic; chmelikoval@vut.cz (L.C.); zumberg@vut.cz (I.Z.); kralikmartin@vut.cz (M.K.)

**Keywords:** touch sensor, touch control, virtual reality, immersive visualization, confocal microscopy, fluorescence microscopy, volumetric data, microscopy images

## Abstract

With the development of light microscopy, it is becoming increasingly easy to obtain detailed multicolor fluorescence volumetric data. The need for their appropriate visualization has become an integral part of fluorescence imaging. Virtual reality (VR) technology provides a new way of visualizing multidimensional image data or models so that the entire 3D structure can be intuitively observed, together with different object features or details on or within the object. With the need for imaging advanced volumetric data, demands for the control of virtual object properties are increasing; this happens especially for multicolor objects obtained by fluorescent microscopy. Existing solutions with universal VR controllers or software-based controllers with the need to define sufficient space for the user to manipulate data in VR are not usable in many practical applications. Therefore, we developed a custom gesture-based VR control system with a custom controller connected to the FluoRender visualization environment. A multitouch sensor disk was used for this purpose. Our control system may be a good choice for easier and more comfortable manipulation of virtual objects and their properties, especially using confocal microscopy, which is the most widely used technique for acquiring volumetric fluorescence data so far.

## 1. Introduction

Light microscopy has played an important role for many years in biological research by enabling observations of a wide range of objects, from cells or other biological samples to macroscopic organisms. Fluorescence microscopy enables the observation of even the smallest structures of organisms in great detail. Individual sections or compartments can be examined by staining them with specific fluorescent labels. This enables the high-resolution acquisition and visualization of multidimensional and multicolor data. Although the raw data in confocal microscopy are 2D images, 3D images can be generated by collecting a sequence of these 2D images and stacking them atop each other. This collection of sections is called a “z-stack” and can be represented as a three-dimensional matrix. The widespread use of fluorescence microscopy has provided motivation for technological improvements and the development of new methods to improve resolution and overcome the limitations associated with light diffraction. Modern super-resolution microscopy methods can achieve a lateral resolution of less than 200 nm and an axial resolution of less than 600 nm. For example, stimulated emission depletion microscopy (STED) [1] achieves a lateral resolution of about 30 nm. This method uses an additional depletion beam, with a higher wavelength than the excitation beam, which has an annular cross section. The resolution is improved by quenching of the fluorescence at the edge of the illuminated area, with fluorescence occurring only in the unquenched area within the annulus. In addition to STED, many other different high-resolution microscopy techniques have been developed, such as reversible saturable optically linear fluorescence transitions (RESOLFTs) [2], structured illumination microscopy (SIM) [3], stochastic optical reconstruction microscopy (STORM) [4], photoactivated localization microscopy (PALM) [5], and fluorescence photoactivation localization microscopy (FPALM) [6].

The acquisition of high-resolution 3D volumetric data is no longer limited to (scanning) confocal microscopy. Light-sheet fluorescence microscopy (LSFM) is another interesting technology for generating 3D fluorescence data. In this modification of wide-field fluorescence surface microscopy, the excitation beam passes perpendicular to the objective. A thin optical section is obtained from the plane of sharpness; the thickness and dimension of the field of view depend on the numerical aperture of the lens used and the depth of focus of the excitation beam. Several planes of the section are gradually acquired and can then be combined in a 3D reconstruction. However, LSFM requires a special hardware component for the laser illumination as an external module to the wide-field microscope [7]. The 3D detailed display is, thus, while increasingly available, quite technically complicated.

Thick samples, such as cells and tissue sections, can present problems for conventional wide-field fluorescence optics. Strong fluorescent signals from objects outside the focal plane result in a low-contrast image. Procedures for processing 3D volumetric data can help compensate for these effects. For example, deconvolution algorithms are used in both confocal and wide-field fluorescence microscopy. In the latter case, a blurred image is a common problem due to the out-of-focus capture of a signal; the blurriness is easily corrected with these algorithms. In wide-field fluorescence microscopy, the combination of the technological capabilities of gradual focusing and obtaining images from several z-planes with the 3D reconstruction from the obtained sequence of images allows a resolution close to that of confocal microscopy. Leica Microscopy’s newly developed Thunder Imager solution offers a simplified implementation of this software analysis without the need for special external hardware [8]. This software system uses a combination of the sequential acquisition of images in different z-positions and the processing of individual images to remove out-of-focus blur through computational cleaning. The results are fast and easily rendered as biologically relevant 3D models. Practically, this means that the use of additional hardware or software solution for obtaining 3D fluorescence data in wide-field microscopy can be helpful to obtain 3D biological reconstruction for further analysis.

Modern microscopic techniques, such as laser scanning confocal microscopy (LSCM), yield 3D and multidimensional data (4D and 5D). Unlike in the 2D case, visualizing 3D and multidimensional data can be a difficult task. Computer screens are exclusively two-dimensional, which makes it difficult to visualize the third dimension of information in an image. The 3D data must be transformed into a single 2D image, and information will always be lost during this step. Most light microscopes give us a 2D view of a physical object. We usually then observe projections of a three-dimensional physical structure onto a two-dimensional surface. This means that one dimension is lost, which significantly limits our perception of the physical reality. Determining three-dimensional structures from the macro to the atomic level is a key focus of current biochemical research. Basic biological processes, such as DNA metabolism, photosynthesis, and protein synthesis, require the coordinated action of many components. Understanding the three-dimensional organization of these components and their detailed atomic structures is essential to interpret their function. Therefore, nowadays, the method for displaying and visualizing the obtained data is an important part of microscopy. Properly designed visualization of volumetric fluorescence data must allow the data to be displayed at any scale and an array of details to be highlighted. There are many open-source imaging applications originally developed to solve the problem of visualizing three-dimensional images. The commonly used ImageJ [9,10] and Fiji [11] can store multiple channels and visualize them in three dimensions using the “hyperstack” function. The Visualization Toolkit (VTK) [12] does not provide a module for visualizing “more than RGB” channels. Thus, with VTK-dependent software tools (OsiriX [13], 3D Slicer [14,15], etc.), it is difficult to fully visualize multichannel fluorescence microscopy data. The main disadvantage of Fiji is that it does not include an adequate tool for the 3D rendering of multichannel data. FluoRender [16] corrects this shortcoming.

Virtual reality no longer is just a part of the gaming industry but is gradually being utilized for scientific applications and integrated into the teaching process. It has a place, for example, in medicine in the simulation of operations, the teaching of anatomy, and the imaging and analysis of biological objects. Another potential use is the visualization of 3D multicolor objects obtained by fluorescence microscopy. Conventionally, 3D fluorescence data are visualized on large monitors or using data projectors. However, now VR technology provides an innovative way to visualize multidimensional image data and models so that a full 3D structure can be understood quickly and intuitively, even at large volumes, with various object parameters or details on or inside the object easily viewable.

A considerable part of the visualization software designed for specialized applications, such as observation and work with biological data, is software based and is mostly used for more advanced work with the object (observation, analysis). Classical (default) drivers and direct interaction of these drivers with individual elements of the visualized menu or with the object are used. These controllers are usually displayed in VR in the form of virtual controllers, for example, Medicalholodeck (medicalholodeck.com; medical virtual reality for 3D surgery planning and education), CellexalVR [17] as a virtual reality platform for the visualization and analysis of single-cell gene expression data, and VR neuron tracer [18]. Some other control systems are based on monitoring hands in space [19]. The hands and fingers are then visualized in a virtual environment and can be used for direct virtual object interactions. As shown in [20], it is also possible to combine the use of a gamepad (for object manipulation) and hand tracking to control other functions.

In 2018, a new software tool, ConfocalVR [21], was introduced. It uses VR systems to completely immerse the user within a 3D cellular image, which he or she can directly manipulate via interactive controls. In this virtual environment, the user can adjust image parameters (contrast, brightness, lighting), work with the histogram and different color channels, rotate the object to observe surface details from different sides, zoom, and even communicate with other users in a shared virtual space (enabling real-time research and discussion). The display process of ConfocalVR consists of several steps. Multidimensional microscope images are first preprocessed in ImageJ and then loaded into ConfocalVR. The user puts on a VR headset and immediately sees the image data as a fully rendered 3D image that can be grabbed and rotated, moved and resized, and visually adjusted using virtual slider controls [21]. Three primary visual rendering modes have been implemented, each providing a unique view of the image structure. The default is a translucent mode. Another, an illuminated mode, uses a virtual external light to create isosurfaces that reflect the boundaries between cellular structures. The last, a cut-range mode, can be used to remove background noise as well as oversaturated voxels from the image. In some cases, this restores important details in the image. ConfocalVR has been replaced by ExMicroVR [22] (free to nonprofits), which newly offers a number of extensions. These include, in particular, a larger range of visual parameters in the menu and the addition of more menu elements (such as checkboxes). Menu items can now be accessed and controlled using a virtual pointer. On the other hand, ConfocalVR is now only available in the commercial version. 

There are several other examples of using virtual reality to display microscopic data. Arivis [23], a provider of software for the visualization, analysis, and management of large image data, announced the new VisionVR [24], which now allows users to view 3D and 4D image volumes and surfaces in an immersive environment. It uses direct volume rendering techniques to display image data in virtual reality, allowing the assignment of each individual data point to the original multidimensional voxel image within the rendered object. VisionVR gives users complete control over how their data are rendered in VR. Using VR controllers, a user can directly rotate, move, scale, shape, mark, classify, measure, edit, and segment their digital image data. The tool also allows a user to save a so-called 360-degree movie, which can be played on a standard desktop computer using a mouse. The disadvantage of this commercial software is its high purchase price.

FluoRender [16] brings new possibilities as an advanced free and open-source software released under the MIT License. It is an interactive tool for multichannel fluorescence microscopy data visualization and analysis. The FluoRender visualization combines the rendering of multichannel volume data and polygon mesh data, with the properties of each dataset independently and quickly adjustable. It is also able to draw and mix channels in different modes, apply 2D image space enhancements, play confocal time sequences, extract structures by painting, and visualize polygon models from these extracted structures along with volumetric data. FluoRender offers two main plot methods for basic plots. The direct volume rendering (DVR) method is very computationally intensive but generates realistic 3D images that reflect the physics of light transmission and absorption. The second method, maximum intensity projection (MIP), is much simpler. This method takes the maximum value of the signal intensities between the voxels that are observed by the observer along the same light path [16].

In addition to the VR visualization itself, there is a need to interface with the virtual environment and objects. Outside of VR, differently complex models, such as those created by CAD and other computer modeling, or visualized medical volumetric data, are commonly displayed and controlled on PCs and computer workstations using available common controls—computer mouse, keyboard, and trackball, which allow the manipulation of the object and control of basic object properties. Other functions are usually accessed via software navigational and functional tools located in visualization or modeling software. There are also special desktop controllers, such as the trackball mouse or SpaceMouse [25], that make it easier to work with model controls. In virtual reality, the control of various virtual object parameters is more complicated due to the absence of visual contact with the controls (software controls and usually also hardware controls). Many free visualization programs (e.g., ImageJ, FluoRender) solve the problem by allowing the connection and use of a keyboard, PC mouse, or gamepad as an auxiliary control for object basic manipulation: it’s rotation and translation in *x*-, *y*-, and *z*-axes or it’s resizing. For controlling of further functions, the user is dependent on switching between VR and reality unless the software environment is visualized directly in VR. Most VR sets (e.g., HTC Vive, Oculus One) are equipped with universal controllers that are designed to handle common uses defined by the entertainment industry. The main controls, such as an index finger trigger or a small joystick or a small circular touchpad, are designed for such applications, where shooting, grabbing, and releasing of objects or moving or teleporting of the user is needed. Additional buttons then allow menu launch and menu item selection to change a virtual object’s features, such as its color or size. The main limitation of common controls for VR systems is the need for sufficient space around the person to allow the manipulation of the controllers both in front of and around the user. This is also associated with a need to monitor the position of the controls in the hands in space with additional hardware, either IR beacons with sensors on the controller (HTC Vive Pro) or VR headsets with a built-in camera system (Oculus One).

When working with virtual objects, a number of operations are performed in the form of “holding” an object in space and moving or rotating it via hand motions. Especially object size can be usually changed by handling the object with both hands and by stretching or pulling the hands. Similarly, when changing an object’s parameters, it is necessary to first grab the object in the space and then select and adjust options from the menu with the help of the controller’s additional buttons. In addition, individual controls, such as buttons on the controller, are difficult to locate in virtual reality. Therefore, a tile-type virtual menu that is visualized in front of the user is more often used to make other controls available. The user can select from the menu using a virtual pointer. However, the need for sufficient space around the user creates a number of constraints (e.g., when observing and manipulating objects in VR in a small-space microscopy lab). This is especially complicated when working with a multiparametric object, such as a visualized fluorescent multicolor sample. The need to handle fluorescent objects is partly solved by ConfocalVR, which offers a large virtual menu with a number of different parameter controls in the form of horizontal sliders. However, there is still a need for strenuous manipulation of the object and menu controls and, therefore, a need to dedicate substantial space. ExMicro and the new (but commercial) version of ConfocalVR make menu control more comfortable when a virtual pointer is used.

Our goal was to create a control system for virtual multidimensional and multicolor microscopic data without the limitations described above. Our gesture-based VR control system is intended to allow manipulations of the object, handle a number of object parameters, and last but not least, reduce hand strain during operations and eliminate the need for substantial clear space in front of the user. Our device, a two-handed controller with a large touch disk at the front, is therefore designed to control virtual objects with only the controls on the controller, eliminating the need for the user to move their hands in space. It is therefore possible to control objects while standing or sitting, without the need to move the body in space. We particularly focused on the manipulation of fluorescent objects acquired using confocal microscopy. For this purpose, the controller was connected to the open-source FluoRender system. FluoRender provides access to many other features of the object and processes to manipulate it, including the ability to slice the object and change its transparency, contrast, and surface type. Our solution is advantageous when used directly at the workplace with a confocal microscopic system, or anywhere at the desk, for example, by students when sharing and viewing virtual objects during their lessons, or, similarly, at professional conferences. The controller with a large touchable disk (multitouch sensor) for gestures with up to five fingers in connection with classic buttons serves as an optimal instrument used to process a wide array of control combinations and handle many actions performed with and on virtual objects.

## 2. Materials and Methods

**Figure 1:**
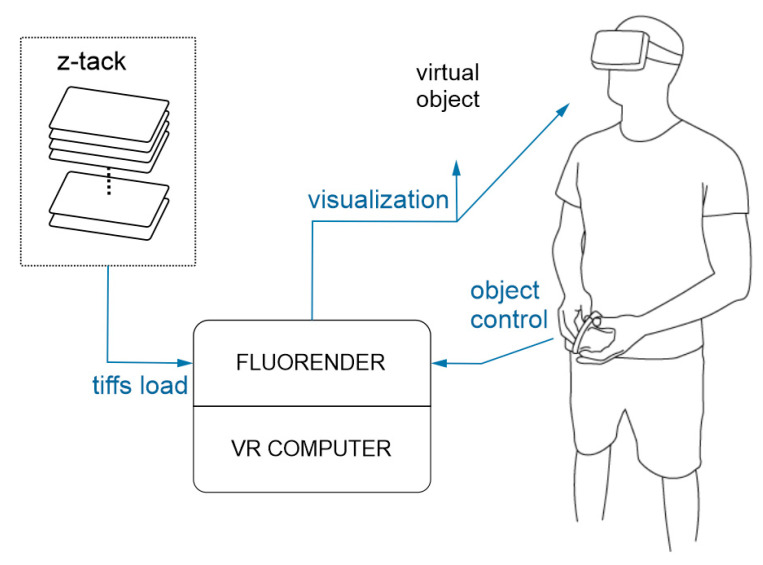
Operation of the complete VR control system with the developed controller. After loading tiffs of the z-stack images with different color channels to FluoRender (installed on a VR computer workstation), the z-stacks are combined and visualized as a complete multicolor virtual object. The instructions sent with the controller are received by FluoRender and translated into actions applied to the virtual object.

The introduced control system is designed to easily handle and observe confocal microscopy 3D models projected in virtual reality (see Figure 1).

**Figure 2:**
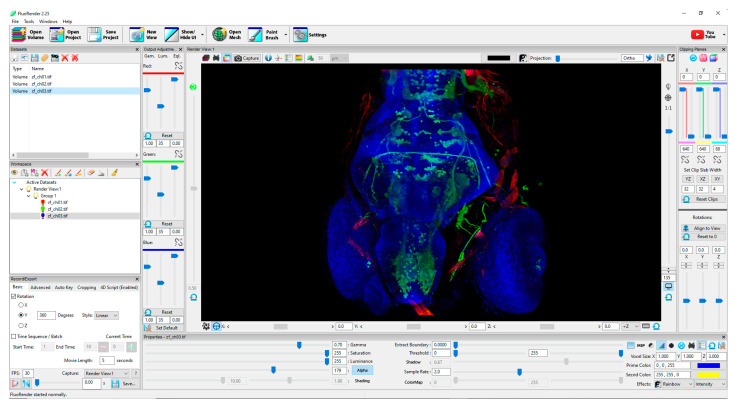
FluoRender with a visualized zebrafish head. Most of the image parameter controls are located in a large menu around the object.

Although most of the controls in FluoRender are available from the menu around the displayed virtual object (see Figure 2), the control method must account for the inability of the user to use these software controls or to look at the physical controls on the controller while observing the model in the virtual environment. Exiting from the virtual environment to work with the visualization software or hardware out of VR is extremely inefficient. The control device is designed to allow easy application of various functions to control a virtual object and its visual parameters without this need. 

The designed control system includes controls for spatial manipulation (spatial move, rotation, zoom, etc.) of the object to get the optimal view, pointing and positioning for observing details on the object surface or inside the object, and can directly control a number of image properties of the virtual 3D objects (e.g., brightness, contrast, color channel transparency and color saturation, and switching off and on color channels) at the same time. It is designed to be ergonomic for being grasped and held for long periods to allow comfortable detailed observing or analyzing of volumetric microscopic data.

**Figure 3:**
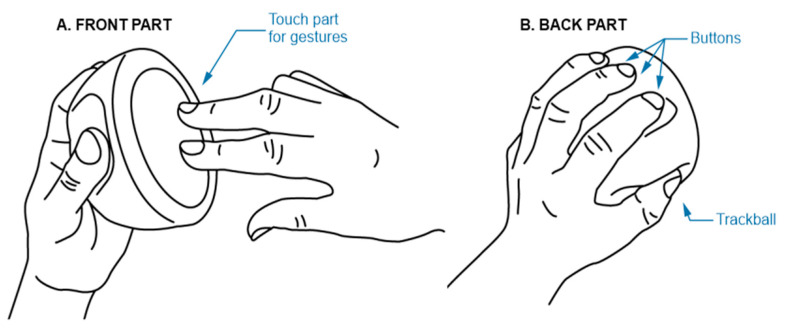
Example of the control panel attachment from (**A**) a front view of the touch disk for the dominant hand’s fingers and from (**B**) the rear with fingers placed in appropriate positions with fingertips directly on the buttons.

To handle a range of operations, the designed device uses a large circular multitouch panel (T201 MTCW, Grayhill, Inc., La Grange, IL, USA) that has several advantages. The touchpad can provide detection of a range of actions performed by one or more fingers, whether single touches, taps, movements, or specific gestures can be used. Suitably positioned in the palm of the hand together with the ergonomic layout of the other controls, it can also be easily perceived and used even with a VR headset on. The control device is designed to be held in both hands. The back and front of the controller contain separate controls for each hand. The dominant hand is used for active control of the touch panel by means of touches and gestures (see Figure 3). The fingertips of the nondominant hand are placed directly on the buttons when the device is held, so it is not necessary to locate the buttons while in the VR space, which is usually difficult to do while using standard controllers. By combining the actions performed on the touchpad and by the buttons, quick engagement of the various functions available in the FluoRender environment can be achieved.

The touch panel has a sufficiently large touch area for manipulation and enables the use of up to 5 fingers at the same time, together with detection of the number of touches and tracing of positions in the *x*–*y* plane for all 5 fingers. By using several fingers, it is possible to perform a wide range of gestures or obtain just specific values by continually moving one or more fingers in the *x*- and/or *y*-axis. The controller is wirelessly connected to the VR computer workstation using a receiver connected via the computer’s USB port (receiving dongle). RF communication with the ESP-NOW protocol is used for transmission. ESP-NOW is a P2P protocol developed by Espressif that enables multiple ESP devices to communicate with one another directly via low-power 2.4 GHz wireless connectivity. It is suitable for battery-powered devices, allowing an ESP32-based microcontroller unit (MCU) to be used as the control board. FluoRender is then supplied with data from the USB dongle via the serial communication. A custom algorithm is implemented in the FluoRender environment, interpreting the incoming instructions to perform actions in the FluoRender environment, which are then projected into the VR environment. Use of the USB dongle as a communication intermediary ensures secure and stable data transfer; the dongle also performs some supporting function initialization settings forwarding.

### 2.1. Navigations and Notifications

**Figure 4:**
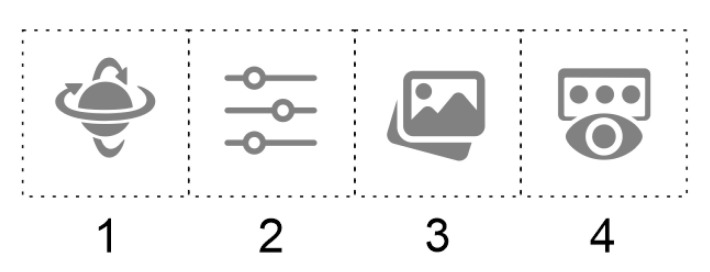
Example of the designed single-line menu with 4 various function categories.

In order to systematically access the individual functions of FluoRender, the functions were sorted into categories by type, with switching enabled between the categories. The user must be aware of the current function category and the other categories offered. For this purpose, a tile menu was created with the icon of the active category highlighted to indicate the current selection. The menu is visualized directly in the FluoRender environment with sufficient contrast to be well visible. An example of the designed tile menu with 4 different categories of functions symbolized by specific icons is shown in Figure 4. The menu is a single line, but in practice, it is expandable by any number of rows and columns. The menu contains the following function categories: 1, object manipulation in space (e.g., rotation, centering, moving, resizing); 2, object clipping (clipping planes) in up to 3 separate color channels, or channel switching; 3, image features (e.g., gamma, luminance, HDR setting); and 4, render modes—layered, depth, or composite mode. 

To prevent the menu display from interfering with the display of the 3D object, the menu is positionally variable—the default position (in front of the user) can be changed to the bottom of the user’s field of view. The controller can be used to display and hide the menu as required.

### 2.2. Tasks Management

The algorithm developed for our VR controller works with support from the SDK provided by the manufacturer of the touch panel (Grayhill, Inc., La Grange, IL, USA). The algorithm provides detection of various tasks performed on the circular touch panel: number of touches, fingers’ X and Y positions, and various actions performed on the buttons (single press, double press, long press). Then, the tasks detected are evaluated in a specific hierarchical order to determine (1) functions group (category), (2) action to be performed, and (3) positional or calculated values. The functions category (a set of available functions) is switched by a special action (e.g., pressing the index finger button). Then the tasks that will be executed in the virtual environment are selected from the operations performed using the controls (on the touch disk or via buttons) (e.g., the detected number of fingers that touch together with the distance between individual fingers). During gesture performance, *x* and *y* coordinates of the fingers are obtained, and the distance between fingers is calculated. The corresponding instructions are transmitted wirelessly to the dongle as a coded sequence of values and passed via serial communication to FluoRender, where they are executed as specific tasks. The 3 buttons were also chosen because the fluorescence data contain 3 color channels (respectively, FluoRender allows you to display and work with 3 separate channels). Each of the buttons can thus be easily used to focus actions on a specific channel (red, green, or blue).

**Figure 5:**
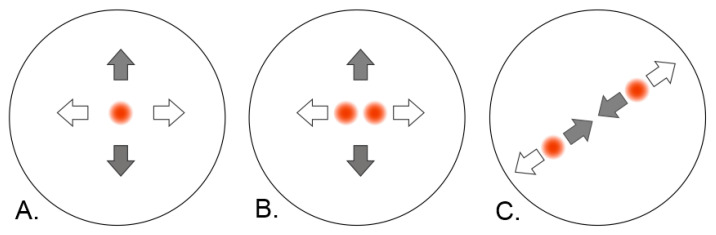
Example of the control for the mode used for an object manipulation in a space. (**A**) Touch and movement with 1 finger provide rotation in a space (by movement in the *x*–*y*-axes), and (**B**) touch and movement with two fingers ensure moving the object in a space (by movement in the *x*–*y*-axes), while the fingers are placed close together. (**C**) Thumb and index finger touch at a sufficient distance more than the threshold value and their movement towards and away from each other will make the object smaller or larger.

A sequence is generated during any action performed using the controls (touching the disk or depressing the buttons). Common instructions associated with operations regarding the 3D object can be replaced by special actions, such as reset (position or values), display, or hide menu. Relative changes in the evaluated finger positions are used with different signs when moving forward and backward on the touch panel. These values are then used to change the value of a specific virtual object visual parameter (e.g., adjusting the contrast and transparency levels or switching the color channels on and off). In the spatial manipulation mode, *x* and *y* values are used to rotate, displace, or scale the object. An example of the application of several different touches and related gestures is shown in the examples in Figure 5.

**Figure 6:**
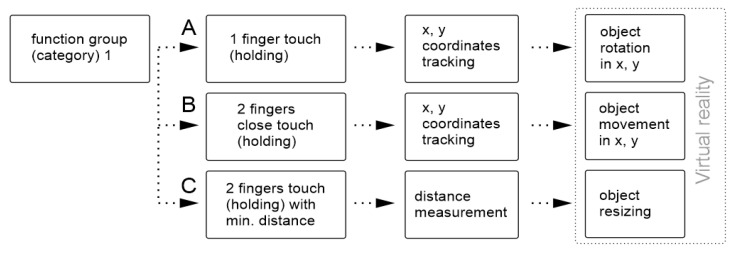
A scheme showing the sequence of the individual operations from Figure 5. After distinguishing the number of touches and the distance between the fingers, the x and y values are traced (**A**,**B**), or the distance between the fingers is measured (**C**).

A scheme showing the sequence of the individual operations from Figure 5 for manipulation of the virtual object (translation, rotation, resizing) is in Figure 6. As can be seen, the operations differ in the number of touches. In the first 2 cases (A,B), changes in the *x* and *y* positions are applied to interact with the virtual object. In the case of changing the size of the object, a touch with 2 fingers must be performed over the preset minimum finger distance, and at each movement, the distance between the fingers is calculated and applied in object resizing. 

### 2.3. Electronics and Functional Design

**Figure 7:**
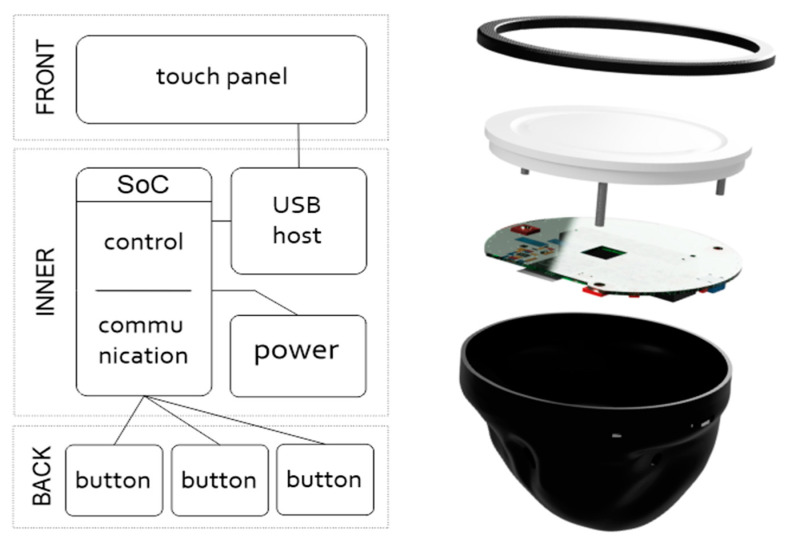
Block diagram of the controller (**left**): the basic control elements are connected directly to the MCU, while the touch panel is connected via a USB hub. The MCU provides power to the peripherals. The VR controller construction (**right**): the controller consists of a rear cover assembly with finger buttons, a cover for the electronic board and rechargeable batteries, and a front-mounted touch disk with an attachment to the rear.

The power supply and control and communications components are integrated on a single-sided user-made electronic control board. The core of the control board is an ESP32 MCU (Espressif Systems); this compact system on a chip enables interfacing with the touch panel via a USB host, detection of events from the controls, processing of values, and wireless communication. In addition, it handles various support functions related to battery charging or power management (see Figure 7). A small USB dongle, including ESP32-PICO-KIT (Espressif Systems), is used in combination with the board to enable ESP-NOW communication.

The touch panel is mounted in a structural sleeve at the front of the controller, to which the rear panel is also attached. The circular electronic control board is mounted behind the touch disk. The indicator LED and USB-C port for charging are located on the side of the board, close to the holes in the back cover of the controller. Another electronic circuit (a button module) with 3 buttons is in the back cover from inside. The MCU is powered by an integrated LiFePO4 battery cell with a capacity of 600 mAh. The smart power system provides indication of battery status, power from the connected USB-C power supply, and simultaneous battery charging. A power saving system is implemented on the device, which is put to sleep to save energy when idle. The device can be switched on by pressing any of the control buttons, rather than using a dedicated on/off switch.

## 3. Results

### 3.1. Practical Results

**Figure 8:**
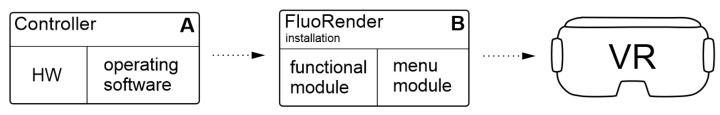
Schematics of two control parts of the control system. The physical controller with the operating software (**A**) and FluoRender installation including two modules for function operating and menu visualization (**B**).

The control system was created as a set consisting of a physical, battery-powered controller, including our custom HW (control board with connected peripherals), custom construction, and ergonomics, as shown in Figure 8A. It includes a control software for handling operations on the controller, with the communication section (see Figure 8A) providing the transmission of coded data toward the second part. 

The second part consists of a functional module for FluoRender installation for interpreting the received data and executing the functions in FluoRender (see Figure 8B). Another module for FluoRender provides tiled menu visualization and control, as menu module in Figure 8B.

The final design of the controller is ergonomic—the back cover easily fits into the palm of the hand while at the same time enabling button presses, as the fingers are guided by ergonomic slots in the cover. The thumb and little finger are placed in the side slots to brace the controller against the palm. The touch disk (multitouch sensor T201 MTCW in white color, Grayhill, Inc., La Grange, IL, USA) was modified; the outer part was trimmed to reduce its size, creating a compact interactive touch panel with a fully utilized surface. The back panel and the front sleeve (a ring) were 3D-printed with an FDM printer. The main cover was printed from a hard plastic material, while the front ring was printed from rubber plastic for easy stretching on the controller from the front side.

**Figure 9:**
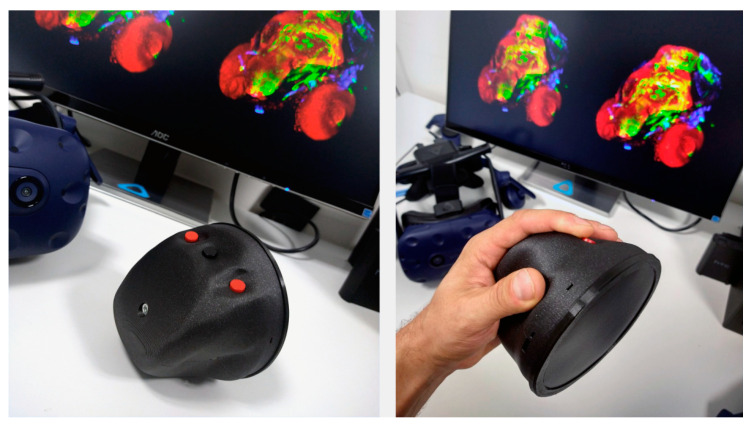
Demonstration of a physical 3D printout with the buttons and the front touch panel.

The first prototype of the controller was prepared and tested in a form of a desktop version including an MCU development kit, MAX3421E-based USB Host Shield 2.0, multitouch sensor (T201 MTCW), and button assembly. Then, for the final design of the controller including our custom-made electronics board, the power supply system, charging and power management functions, and communication were first verified. Then the control functions were tested. A desktop VR computer workstation equipped with an HTC Vive VR kit or Lenovo Legion VR laptop equipped with Microsoft Oculus was used for the tests. The final version of the controller with the ergonomic electronics enclosure including three buttons and the touch panel covered with black foil in the front part is shown in Figure 9. LED indicators and an input for the USB-C connector near the thumb can be seen in the cover. In the background, FluoRender running on stereo mode and the HTC Vive on which the controller was tested can be seen.

**Figure 10:**
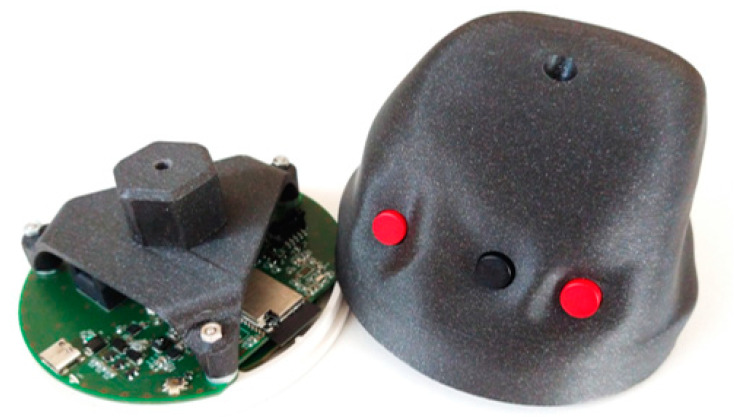
Demonstration of the electronic control board of the controller with a battery holder and cable connectors for connection to the button module.

The construction was prepared to make the controller easier to be assembled or opened. The real construction including the touch disk with a connected electronic board is shown in Figure 10. The circuit board contains three holes near its edges, which are used to push the board onto the back of the touch disk. There is a special 3D-printed holder connected to the front part of the controller. That enables the controller’s front and back parts to be fastened together using a single screw and makes it easy to open. There is an especially shaped input for the holder inside the rear cover of the controller. The hole for the screw in both parts can be seen on the top.

**Figure 11:**
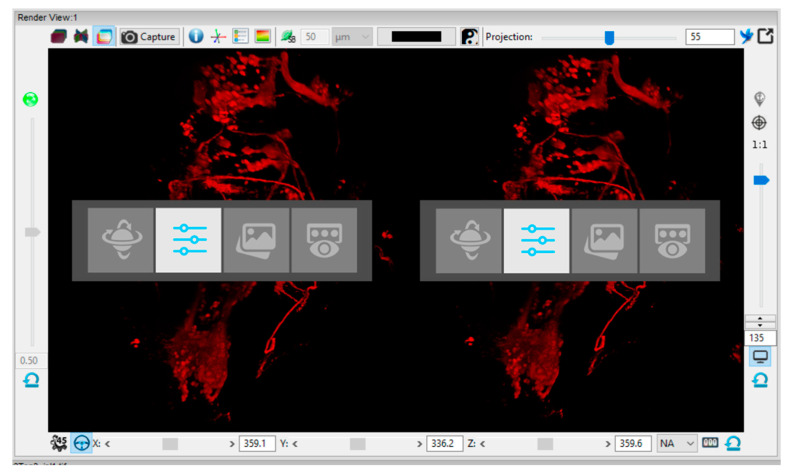
Example of the displayed tiled menu on stereo mode (for VR). The second mode for object cropping and color channel control is active.

The functional menu was prepared as shown in Figure 11. During the menu tests, minor problems were found with its visualization on the Oculus One VR device. When the hardware was being overloaded, unsynchronized frame changes that resulted in occasional menu flickering appeared. Therefore, an alternative version of the menu has been prepared. The alternative menu includes only one tile in the menu, and the category icons are iterated in that one position. It helped to reduce the mentioned negative effect. Finally, it can be switched between these two different versions of the menu in the text settings file, which is a part of the control system files. 

The controller went through a series of optimization processes during testing. The touch controls were software-optimized for accurate evaluations by holding positional data during the gestures into a buffer, which is used for signal smoothing. Range and sensitivity settings for the controller were added, and they can be set in the settings file.

### 3.2. Validation in a User Study

Comparison with other control methods was prepared in the form of a user study, with two different application cases. A set of steps typical of volumetric data observations performed by a biological researcher was prepared. Designed operations are typical for observing a large biological object (Case 1) or flat biological 3D data, such as cells and tissue (Case 2). The case study is based on the usual need to prepare a virtual object into a suitable form (position, rotation, size) for observation in VR. Then individual processing for close observation of surface or interior details in different color channels is followed. 

#### 3.2.1. The First Application Case (Case 1)

**Figure 12:**
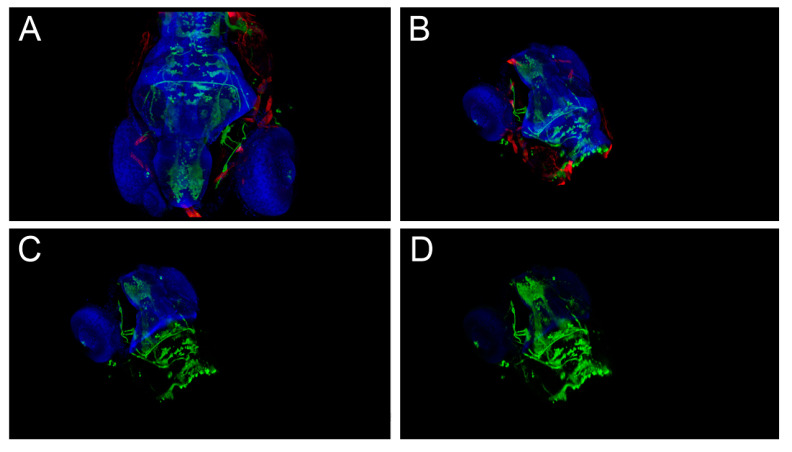
Example of 4 different states of the model reached by steps 1–10 according to the prepared scenario. (**A**) Step 1 from the scenario. (**B**) Object manipulation, steps 2–4. (**C**) Object cropping and switching of the red channel, steps 5–8. (**D**) The result after steps 9-10 is shown. For better illustration, a black background was used, and the stereo mode for VR was switched off when preparing the screenshots. The images were prepared in FluoRender.

In the first case, a sample dataset from the FluoRender website was used. These sample data (see Figure 12A) include a 5-day-old transgenic zebrafish larva, stained for anti-actin, anti-GFP (recognizing the isl1:GFP transgene), and Topro3 (nuclear stain). Anti-actin (in the red channel), or actin antibody, was used to visualize actin structures in cells. Anti-GFP (in the green channel) is a green fluorescent protein antibody, which is used as a valuable reporter molecule for the visualization of the isl1:GFP transgene expression. Lastly, Topro3 (in the blue channel) is a highly specific, sensitive, and stable fluorescent DNA dye that shows specific staining of nuclei without any staining of the cytoplasm. 

The main aim was to observe the details of the internal structure present in the green channel and its inter-relationship with the external structure (cover) present in the blue channel. Thus, the red channel was switched off before working with the structures in the green and blue channels. Then, the visibility of the currently visible structures was enhanced by cropping their parts in both the green and blue channels and by changing the contrast or the visibility.

The individual steps to achieve the goal would be as follows:

1. Loading and opening a model;
2. Resizing the model;
3. Moving the model to the side in the x- and y-axes;
4. Rotating the model in the x-, y-, and z-axes until it is in the appropriate position;
5. Switching off the unwanted channel (red channel);
6. Cropping the green channel from one side;
7. Cropping the blue channel from the same side but for a longer distance (so the inner part in the green channel protrudes);
8. Cropping the green and blue channels together from the top;
9. Making the blue channel (cover) partly transparent;
10. Increasing the contrast in the green channel (inner); and
11. Rotating (in small angles) for a detailed observation of the object.

After loading the data (see Figure 12A), it was first necessary to resize the object to a suitable size for effective observing in VR, which normally differs from the common size of the object when loaded. Then the object was moved and rotated to the specific *x*, *y*, and *z* angles to get to a more effective viewing position (see Figure 12B). The red channel was switched off, and the object was clipped from two sides in each visible channel (Figure 12C) by using six different clipping planes (*x*, *y*, and *z* from both sides) that are available for this purpose in FluoRender. The luminance in the blue channel was decreased, and gamma in the green channel was increased (Figure 12D). Now, a detailed observation of the inner parts of the object can be followed (step 11).

The individual tasks were performed using the HTC Vive Pro VR system with the native controllers in ConfocalVR, ExMicro, and FluoRender using our custom controller. Step 11 was not described in the further list of steps, as it performed the action included in step 4, and the user can return to this action to observe the object. The individual control systems are identified as ConfocalVR [21], ExMicroVR [22], and Gesture Based (for our custom gesture-based controller).

In ConfocalVR, grabbing of the object can be achieved after getting closer to the object, touching it, and pressing and holding the trigger button on the controller. This procedure had to be performed in steps 2–4 before the object was manipulated. It is necessary to use a controller in a similar way when working with any control element in ConfocalVR. This can be performed by both controllers at the same time too. Loading datasets in the first step can be performed directly in the virtual environment after getting closer to the virtual desktop. For switching individual channels on and off, three buttons (R, G, and B) are present on the virtual controller. The buttons are located on the touch disk of the physical controller and must be pressed (touched) here. 

In ExMicroVR, unlike ConfocalVR, the laser pointer is used for focusing on the object or on the controls. Therefore, to manipulate the object or controls, it must be first pointed to it by the laser pointers of one or both controllers. Then, the object is grabbed by pressing and holding the trigger button. This eliminates the need to move closer to the object or to the controls. 

When using the Gesture Based controller (with FluoRender), the tasks were performed seated as it was more comfortable, and there was no need to be standing. To move between the different function categories, button 1 was always pressed (long click, for more than 1 second) to switch between the function categories (according to Section 2.1) before starting step 5 and again before performing step 9. A tile menu appeared in the virtual environment, and the current active icon was changed to the following icon. 

#### 3.2.2. The Second Application Case (Case 2)

**Figure 13:**
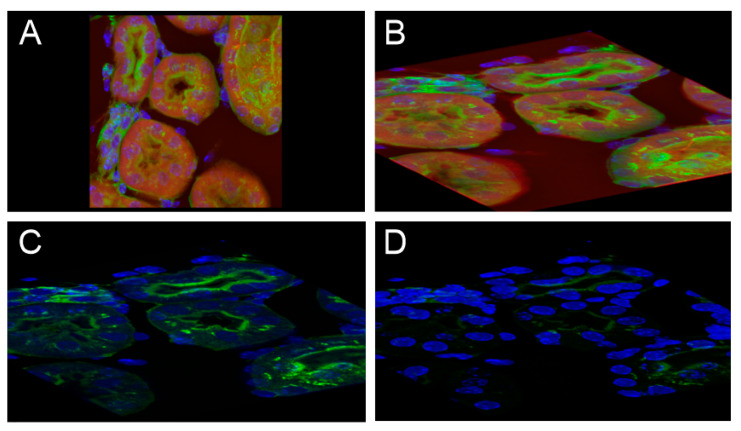
Mouse kidney stained with 3 different dyes (elements of the glomeruli and convoluted tubules by Alexa Fluor^®^ 488, the filamentous actin prevalent in glomeruli, and the brush border labeled by red fluorescent Alexa Fluor^®^ 568 phalloidin, nuclei labeled by DAPI). Images (**A**–**D**) demonstrate similar processing as in Figure 12; however, for (**C**) no object cropping was applied.

The second test case was executed with a prepared slide with a fixed 16 µm cryostat section of a mouse kidney. The scenario from Case 1 was modified and used. Modification was necessary due to the different object’s characteristics. The object is of small thickness compared with its other dimensions, so its rotation and resizing must be performed appropriately. Even the control needs and the time spent on each task can vary considerably. On the other hand, cropping operations applied to the object are not useful now and were skipped. Therefore, only steps 1–6 and 9–10 of Case 1 are included in the modified scenario C2. The results of the applied procedures are shown in Figure 13. 

### 3.3. Results Validation

The individual actions performed by each control system to execute the scenario are shown in Table A1 for both user cases C1 and C2. The individual tasks were evaluated by determining a score from 1 to 5 with respect to the speed of executing the task or the usability of the control and also with respect to the constraints in performing the steps. We also considered the need for longer time or more attempts when correctly grasping the object for ConfocalVR or sometimes retargeting it with the laser pointer and ExMicroVR. We would like to point out that the times for each task could not be easily measured, so any rating is only an estimate and is individual and subjective.

We also faced some limitations with each controller, which affected the scores given. Here are examples of some specific steps and limitations: The score of Gesture Based was reduced for step 4 due to a retouch that is required for *z*-axis rotation in case *x*–*y* rotation is being performed. Additionally, *z*-axis rotation is not comfortable because two fingers must touch at the minimum distance between them and then must be rotated. Rotation must be usually repeated to achieve the required angle. For ConfocalVR and ExMicroVR, on the other hand, rotation poses the problem of needing to rotate the wrist in all axes, which can be quite challenging and limits the range of rotation angles. To achieve the desired angle, the object must be released and regrasped after releasing the wrist. When switching off the visibility of the red channel in ConfocalVR, a virtual button must be pressed. Usually, it was not easy to get the position of the button on the touch circular panel and press it. If any of the steps could not be achieved (e.g., the control system did not allow it to be executed by the system or controller), the step was evaluated as zero. On the other hand, with ExMicroVR it was not easy to hit the checkbox with the laser pointer, and sometimes multiple attempts were needed to reach it.

**Figure 14:**
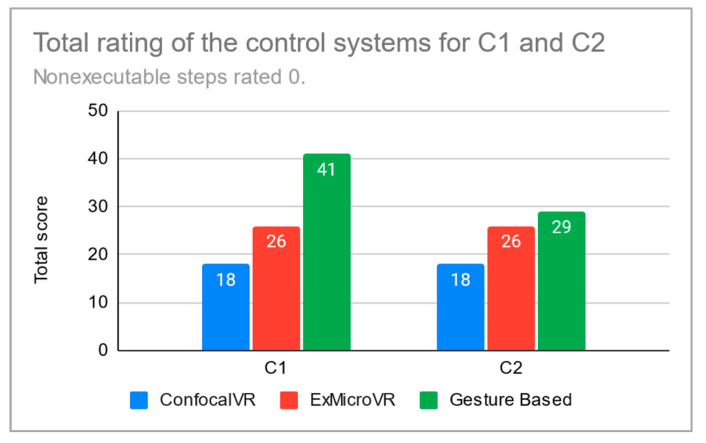
Total rating of the control systems for the application cases C1 and C2. The steps that could not be completed were rated 0.

**Figure 15:**
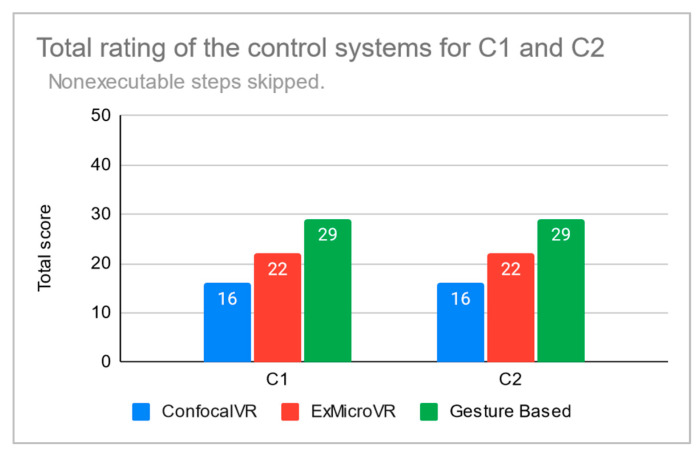
Total rating of the control systems for the application cases C1 and C2. The steps that could not be completed were not rated (rating was skipped for these steps).

Detailed scoring is given in Table A1. In conclusion, the overall scores for the application cases C1 and C2 are shown in Figure 14 and Figure 15. The total score (as the sum of the scores for the cases C1 and C2) is in Table 1. As already mentioned, the scores can be considered the result of our subjective evaluation.

## 4. Discussion

As shown in the case studies, a classical object manipulation method requires considerable manipulation in space. This mostly involves the actions of moving closer to the object, grasping the object after extending the hands, and moving with one or two controllers in space. This can be considered a major disadvantage when handling confocal data in a small space laboratory, in a small room, or at a desk. Moreover, achieving *z*-axis rotation cannot be easily achieved as it is limited by the reduced mobility of the wrist of the hand, whereas *x*- and *y*-axis rotation can be achieved more easily but only with limited tilt angles. Similar limitations can be expected for hand-tracing systems. This is partly eliminated by ExMicroVR, where only some workspace in front of the user (up to the distance of the outstretched arm) is required to use the controllers as laser pointers properly. Another issue relates to grabbing the controlled object when manipulated with it. The object needs to be held continuously. This causes small movements of the object (due to hand tremors when holding it) and makes it impossible to perform this task for a long time. On the other hand, our controller allows for alternating the observations of the object with the actions of manipulating the object. Additionally, a movement in *x*, *y*, and *z* is simpler and more comfortable. Movement is limited only by the space on the touch disk. When the movement limit on the disk is reached, the fingers can be put back and the action (e.g., *x* rotation) can be continued. Many other various specifics and limitations of the compared control systems have already been mentioned in the Results section. 

One of the most advanced and challenging tasks is to crop objects in three different axes from both sides in each axis. In the ConfocalVR and ExMicroVR software, cropping of individual object channels could not be achieved separately. These SWs offer the possibility to crop the object only as a whole using the so-called Excluder, which allows moving into and looking around inside the virtual object. Then, some parts of the whole object can be hidden. The resulting effect is different from the use of object cropping in FluoRender. In FluoRender, clipping planes are used for this purpose, and the object can be clipped in all three (*x*-, *y*-, and *z*-) axes from both sides, yet separately in all channels. This brings significant advantages, and our controller is adapted to these features. However, the operation of these functions is complex, and even in our case, the control was not set up to provide a comfortable operation, as it consists of several different steps that the user must apply. The current solution needs to be optimized for better handling of these functions in the future.

It should be noted that a gamepad can be used in FluoRender to control the object. However, the gamepad has limited capabilities and only handles spatial manipulation of the object. Even with the potential mapping of additional elements, the number of functions that could be controlled would be limited, or the function buttons would be hard to access when VR glasses are on. Therefore, the gamepad tends to be used in VR specifically with the aim of moving an object.

The presented gesture-based control system has some other benefits. It allows a virtual object to be manipulated independently of the additional VR hardware equipment, such as headset cameras or IR emitters and sensors that are usually used to sense the spatial position of the classic controller. Control instructions are transferred from the gesture-based controller to the FluoRender PC installation with which the device communicates, while FluoRender performs the visualization. Therefore, the hardware is dependent only on FluoRender’s requirements. On the other hand, the introduced control system is incompatible with VR systems that require free hands or fingers for special VR actions, because both hands are fully used and unavailable for any further operations. However, the control system was designed only for fast and comfortable observation of the virtual multidimensional and multicolor object. It is not intended for further detailed object analysis. 

## 5. Conclusions

In the examples and results, it has been shown that using a combination of a circular multitouch disk and three buttons can be a practical way for effective control of the basic multichannel confocal 3D data in virtual reality. It has been shown that some tasks can be performed very quickly and comfortably, such as switching the visibility of individual channels off and on by double-tapping or turning the rotation of an object in *x*- and *y*-axes into a movement of the object just by the replacement of two fingers with one in operations. 

FluoRender includes plenty of features, and only some of the functions were mapped. We have selected such functions that are among the most widely used to demonstrate the functionality and potential of our control system. It is assumed that a combination of buttons (short or long press), touch and multitouch by different numbers of fingers, finger distance measurement, and finger tracing options on the touch disk will allow a variety of functions to be mapped in FluoRender in the future. It is also possible to extend the tile menu by a suitable number of rows and columns and possibly use another available way of navigation—by tracing the position in the menu by moving the fingers on the touch panel. 

Although the control system is in current version dependent on FluoRender, it is modular and flexible enough and can be extended and connected to any other visualization system (virtual system) open to external control hardware after an appropriate update in the future.

The control system was developed as an output of a project supported by the Technology Agency of the Czech Republic. The project will be further developed, and the control system is planned to be demonstrated to a professional audience and tested by local confocal microscope users. The use of the controller can be extended to applications where there is a need to manipulate a virtual model and its visual parameters or to observe the model in detail. This and other practical applications are planned to be tested in the future.

## Figures and Tables

**Table 1:** Total score as the sum of the scores for the cases C1 and C2. In the first row, there are total scores for the case in which steps that could not be executed were rated 0. In the second row, the rating of these steps was skipped, so the total score is lower.

ConfocalVR	ExMicroVR	Gesture Based
36	52	70
32	44	58

**Figure A1:**
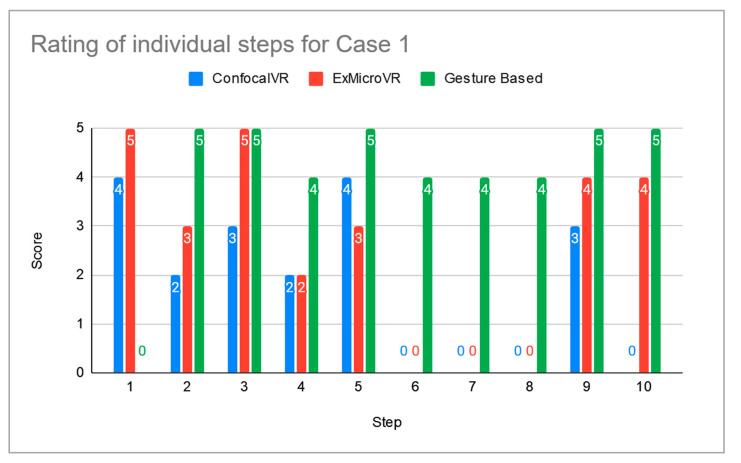
Rating of individual steps for Application Case 1 (C1).

**Figure A2:**
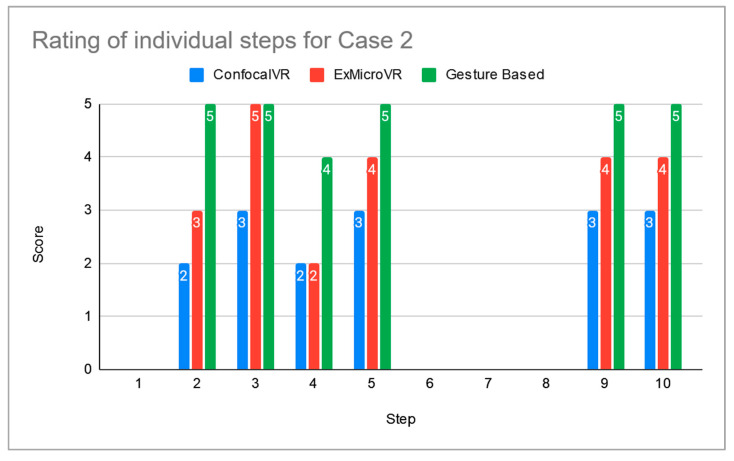
Rating of individual steps for Application Case 2 (C2). In this case, steps 6–8 of the proposed scenario were skipped for all control systems (so they are missing in the graph).

## Data Availability

The datasets generated and/or analyzed during the current study are available from the corresponding author on reasonable request.

## Appendix A

In the appendix, we provide a complete table of the tasks that had to be performed with each control system to complete the proposed scenario. The table is complemented by two figures with a subjective evaluation of tasks using a score ranging from 1 to 5 (5 being the highest). The first figure (Figure A1) contains the evaluation after the execution of scenario 1 and the second figure (Figure A2) after the execution of scenario 2.

**Table A1:** Performed operations for each control system and their rating for 2 user studies in the C1 and C2 columns.

No.	Operation	ConfocalVR	C1	C2	ExMicroVR	C1	C2	Gesture Based	C1	C2
1	Loading	The load image button was pressed using the trigger button to open the file explorer, which helped to select partial data. The individual channels were loaded one by one.	4	4	Like ConfocalVR, the individual channels were loaded one by one using the file explorer and now with the help of the virtual laser pointer.	5	5	The data were quickly loaded in FluoRender before entering into VR (Figure 12A). Note: Our system does not have a virtual folder function to view and open datasets.	0	0
2	Resizing	The object was grabbed with both hands together and moving toward and away from each other (i.e., changing the distance of the controllers from each other).	2	2	Grabbing of the object with both hands together and moving toward and away from each other (i.e., changing the distance of the controllers from each other).	3	3	By placing two fingers (thumb and index finger) at efficient distance from each other and then pulling them slightly together, the object was being reduced in size.	5	5
3	Moving	The object was first grabbed by one hand. The controller was moved laterally (in the *x*- and *y*-axes) while holding the object.	3	3	The object was first grabbed by one hand. The controller was moved laterally (in the *x*- and *y*-axes) while holding the object.	5	5	By touching the disk with two fingers close together and moving in the *x*- and *y*-axes, movement of the object in space was achieved.	5	5
4	Rotation	The rotation of the object was performed by rotating the wrist when grabbing the object by one hand.	2	2	The rotation of the object was performed by rotating the wrist when grabbing the object by one hand.	2	2	Rotation of the object was managed by moving one finger in the *x*- and *y*-axes along the touch disk, alternating with two fingers touching with some distance between and rotating them left and right (for the *z* value decreasing or increasing).	4	4
5	Switching off (R)	The unwanted channel was turned off using the left controller, using one of the three virtual buttons (R).	4	4	The red channel was disabled, unchecking the red channel from the visible column of the checkbox by pointing the laser pointer to the box and pressing the trigger button.	3	3	The red channel was disabled using a double tap by one finger on the touch disk ^1^.	5	5
6	Clipping (G)	The channels could not be cropped separately in the *x*-, *y*-, or *z*-axis, so steps 6–8 could not be achieved.	0	-	The channels could not be cropped separately in the *x*-, *y*-, or *z*-axis, so steps 6–8 could not be achieved.	0	-	Button 2 pressed ^3^. By touching the disk with the index finger and moving to the right (increasing the *x* value), the left clipping plane in the *x*-axis was used and moved to the right (blue channel cropped from the right).	4	-
7	Clipping (B)		0	-		0	-	Button 3 pressed. An identical action was taken for the blue channel.	4	-
8	Clipping (G + B)		0	-		0	-	Button 3 pressed again ^2^. One tap with three fingers was used to activate the *z* clipping plane control. By touching and moving the index finger to the left, the top clipping plane was going lower.	4	-
9	Visibility (B)	A slider in the color filters section was used to increase the contrast, and the slider ball was grabbed and moved to the right.	3	3	The laser pointer and the trigger button were used to uncheck the appropriate menu checkbox in the focus column (for the red channel).	4	4	Button 3 pressed ^3^. Luminance reduction in the blue channel was achieved by placing two fingers on the touch disk and moving it slightly to the left.	5	5
10	Contrast (G)	Contrast could not be enhanced in an individual channel.	0	0	Then, the brightness and opacity properties were changed by sequential grabbing and sliding these sliders using the laser pointer.	4	4	Button 2 pressed. The increase in contrast was achieved by placing thumb and index finger at a specific distance from each other and then pulling them slightly apart.	5	5

^1^ Two or three fingers would be used to hide or make visible the second (G) or third channel (B). ^2^ Actions are active again for the whole objects—for all visible channels. ^3^ Button-pressing means that the further applied functions were later focused on a specific channel so that, for example, if button 3 is pressed, the following actions are applied to the third channel (B). By default, the functions are applied to the whole object (to all displayed channels together). To return to the default mode, it is necessary to press the same button that was used to focus on a specific channel.
